# CTSA Search Solutions: A novel approach to searching CTSA hub website content

**DOI:** 10.1017/cts.2022.485

**Published:** 2022-11-10

**Authors:** Barbara Tafuto, Riddhi Vyas, Trish Pruis

**Affiliations:** 1 Department of Health Informatics, Rutgers School of Health Professions, Newark, NJ, USA; 2 New Jersey Alliance for Clinical and Translational Science, New Brunswick, NJ, USA; 3 Oregon Clinical and Translational Research Institute, Oregon Health and Science University, Portland, OR, USA

**Keywords:** Website, social media, content development, knowledge management, CTSA, NCATS

## Abstract

Clinical and Translational Science Award (CTSA) hub websites are a critical communication gateway to assist the clinical and translational science community and promote CTSA hub offerings. The objective of this funded pilot project was to create a website and online database for the CTSA consortium that allows users to conduct structured searches among the 50 + CTSA hub websites. The result is CTSA Search Solutions, an online, searchable database that includes access to 50 + CTSA hub websites with 80+ structured search term options and over 800 links collected, organized, and published. Hubs can be searched by name and filtered by a specific CTSA topic, state, region, or even number of years funded to make detailed comparisons with the data identified. The home page for each hub can be accessed directly from the search page. The CTSA Search Solutions online database will allow for a wide breadth of CTSA personnel (core leads, researchers, administrators, communicators, and evaluators) to find consolidated information to learn about specific CTSA hub program highlights, as well as conduct research into program hub outputs and best practices across the nationwide CTSA consortium.

## Introduction

In 2006, the National Institute of Health (NIH) created the Clinical and Translational Science Awards to provide resources to clinical and translational science researchers. Between 2014 and 2019, the NIH, through the National Center for the Advancement of Translational Science (NCATS), awarded approximately $3 billion to US Academic Medical Centers to build a national network of program hubs that serve to meet key NCATS goals and initiatives. Today, 50 + Clinical and Translational Science Award (CTSA) program hubs form this network [1].

Each CTSA hub has its own distinct program directives and practices to achieve NCATS goals within the context of their institutional needs and opportunities. Each CTSA program hub has a corresponding website that highlights its clinical and translational science centered programs. CTSA hub websites are a critical communication gateway to assist the clinical and translational science community and promote CTSA hub offerings. The 50 + CTSA hub websites present unique approaches to the five NCATS goals: (1) collaboration and engagement; (2) workforce development; (3) clinical and translational research methods and processes; (4) the special and underserved populations across the lifespan of research; and (5) cutting-edge informatics [2].

CTSA Hub websites are useful beyond individual institutional needs. Exploration and understanding of the activities of other members of the CTSA consortium is useful information for a variety of reasons. Researchers search other CTSA hub websites to learn about best practices, program offerings, basic website presentation, and to gauge clinical and translational trending topics and offerings [3–9]. CTSA hub website developers investigate other CTSA websites for content and design ideas. The Center for Leading Innovation and Collaboration (CLIC) recognized these needs by providing a CTSA Hub Website Directory [10].

The easiest procedure for viewing CTSA hub websites and specific content topics has been to identify the list of CTSA hubs from the CLIC Program Directory Page, then use internet searching to navigate through the many site pages for the content needed [10]. The CLIC Directory page is an excellent starting point for such a task, but it does not include expanded content beyond the home pages for each hub. Searching CTSA websites using this method can be a time-consuming process. A database that presents CTSA hub website content in a structured, searchable format would improve efficiency and help to validate such a search process. A New Jersey Alliance for Clinical and Translational Science (NJ ACTS) Pilot Grant funded an initiative to fill this gap by creating a prototype database that streamlines CTSA hub website searching and maximizes potential benefits of the easier access to website information. The overall goal of this project was to build an end user, searchable database inclusive of all CTSA hubs that organizes and maintains structured content offered by the CTSA hub websites.

## Methods

Four aims were developed to achieve the goals for this pilot project. The first aim was to identify structured search terms related to three general themes: (1) NCATS goals for CTSA programs; (2) CTSA initiatives and common cores; and (3) a “hot topic” in clinical and translational research. The second aim was to systematically identify, collect, and store CTSA website uniform resource locators (URLs) for pages that contain content that align with the structured search terms identified in Aim 1. The third aim was to create, preserve, and disseminate the aligned database content through structured search capabilities within a website that enables end users to conduct simple searches related to their research needs. The fourth aim was to assess the potential impacts of the database on the CTSA community through targeted beta testing.

### Aim 1: Identification of Structured Search Terms

The CTSA Search Solutions team convened in the first month of the pilot award, which was awarded in 2020, to identify structured terms for the database’s general search capabilities. A comprehensive list of CTSA hubs was identified using the NIH Reporter for the year 2020 in combination with the CLIC Directory, as posted on their website in 2021. CTSA hub home pages were pulled from the CLIC Directory or searched for using Google if the home page was not available in the CLIC Directory. Once a comprehensive list of hubs and affiliated home pages was established, then each CTSA hub website was searched and reviewed by the lead investigator for common terms related to NCATS goals, CTSA initiatives and common cores, and a “hot topic” in clinical and translational research. It is important to note that U54 model from 2020 was used to identify NCATS goals and common cores, since, at the time of the funding of this pilot award, the 2021 UM1 model had not yet been rolled out to the CTSA hubs. The first two topics were chosen, because NCATS models its mission, priorities, and funding mechanisms on its goals, initiatives, and common cores. As such, these should be common topics represented on hub websites across the network. The “hot topic” chosen to explore was COVID-19 activities, since this project began in 2020, shortly after the onset of the COVID-19 global pandemic. Collectively, these three topics reflected both the mission and priorities of NCATS at the time this pilot project was initiated, as well as commonalities that should exist across the CTSA hub network.

Once the review process was completed and a list of search terms was compiled, co-investigators independently reviewed terms, flagged any questions or comments in a shared document, and then discussed and came to a consensus on categories, new additions, or changes to search term recommendations. There was very little disagreement among the three co-investigators about the categorization for the first two topics, which was due to the clear and readily available NCATS definitions. However, upon discussion, the COVID-19 activities topic was expanded and further subdivided to include news, funding, research, community information pages, and vaccine info. This was done to capture the breadth of CTSA hub involvement in this area.

Inclusion topics for pages related to NCATS goals for CTSA activities were organized into five categories: (1) *workforce development*, defined as content that supports training and cultivating the translational science workforce; (2) *collaboration and engagement,* defined as content that engages patients and communities in all phases of the translational process; (3) *special populations*, defined as content that promotes the integration of underserved or special populations in translational research throughout the human life span; (4) *methods and processes*, defined as content designed to promote innovating processes to increase the efficiency and quality of translational research; and (5) *informatics*, defined as content intended to advance the utilization of cutting-edge informatics [11].

CTSA hub initiatives and common cores included the most common topics identified among the hub web pages that did not include the NCATS goals category. The inclusion categories for the CTSA hub initiatives and common cores website content were: (1) biostatistics, epidemiology, and research design (BERD); (2) CTSA hub events calendars; (3) CTSA evaluation or impact; (4) CTSA news; (5) KL2 programs; (6) TL1 programs; and (7) pilot awards.

One subcategory of terms was developed related to clinical and translational science education and training offerings identified within each CTSA hub website. These categories included website content related to: (1) basic training (<2 weeks); (2) community engagement training (community related activities); (3) experiential training (trainee has off sight opportunities); (4) scholar and certificate programs; and (5) degree and post-graduate programs.

A final set of terms were identified to help users broaden or narrow their search parameters. These structured terms were developed to serve only as search filters for the database and included three main categories: (1) region, which included six regions of the USA: Midwest, Northeast, Pacific, Rocky Mountains, Southeast, Southeast; (2) states, which included all 50 US states; and (3) number of years funded, defined by the cumulative number of years that a CTSA hub has received funds from NCATS. This parameter ranged from 1 to 10+ years. This information was pulled from the CLIC Directory, which is based on the NIH Reporter. One challenge of using this data source is that the NIH Reporter does not have publicly available data as early as 2006, when the CTSA program funding started. As such, number of years funded only goes back to 2013. These three categories were chosen based on existing delineations among hubs that would lend to useful comparisons. For example, a hub in the Midwest might be interested in seeing how other hubs represent their Community programs in that same region, or a newly funded hub might be interested in seeing how a mature hub organizes its website and which programs it offers.

The search terms and categories were formally defined for the URL identification and collection phase. See Table 1 for detailed definitions of each term and category.

**Table 1.** Structured search terms and definitions

Category and terms	Justification and definition
COVID-19 activities	This section is to help identify how CTSA program hubs are leveraging their vast expertise, resources, and community connections to find COVID-19 answers and improve health outcomes.
Main page	The main page represents a primary page that a CTSA hub has dedicated to COVID-19 activity. It can include a diverse number of topics on COVID-19 and provide links to COVID − 19 sub-categories.
COVID-19 research	This phrase is intended to identify content a CTSA hub has dedicated to any information on COVID-19 research activities.
Funding	This term under the COVID-19 heading refers to COVID-19 funding information a CTSA hub has included on their website.
In the community	This phrase under the COVID-19 heading refers to content a CTSA hub has dedicated to COVID-19 community outreach.
In the news	This phrase under the COVID-19 heading refers to content a CTSA hub has dedicated to COVID-19 news.
Vaccine information	This phrase under the COVID-19 heading refers to content a CTSA hub has dedicated to COVID-19 vaccine information.
NCATS goals	This section focuses on the five goals outlined by NCATS for the recipients of the Clinical and Translational Science Awards.
Collaboration and engagement	This phrase refers to NCATS Goal #2, defined as engaging patients and communities in every phase of the clinical and translational process. It can be defined by hubs by several terms such as Community Engagement, Collaboration and Engagement, and Community.
Informatics	This term refers to NCATS Goal #5, defined as advancing the use of cutting-edge informatics in clinical and translational science. It can be identified by hubs as Informatics or Bioinformatics.
Methods and processes	This phrase refers to the NCATS Goal #4, defined as innovating processes to increase quality and efficiency of clinical and translational science, particularly multisite trials. It can be defined by hubs as Regulatory, Resources, or Research Resources.
Special populations	This phrase refers to NCATS Goal #3, defined as promoting the integration of special and underserved populations in clinical and translational science across the human lifespan. It can be defined by hubs as Special Populations, Underserved Communities, Integrating Special Populations.
Workforce development	This phrase refers to NCATS Goal #1, defined as training and cultivating the clinical and translational science workforce. It can be defined by hubs as Workforce Development, Training and Education, Training, or Workforce.
Additional topics	This section is dedicated to terms and topics that are ancillary to the NCATS goals.
BERD	This acronym represents how hubs present information or services for Biostatistics, Epidemiology, and Research Design.
Calendar (events)	These terms identify how hubs present their monthly events and activities.
Evaluation (impact)	These terms identify how hubs measure their program activities and demonstrate progress toward program goals, including improvements in research translation and workforce development.
In the news	Under the Additional Topics heading, this phrase identifies how hubs present relevant news stories.
KL2	This abbreviation is an NIH-funded award that supports newly trained clinicians appointed by an institution for activities related to the development of a successful clinical and translational science career. Since a CTSA program hub is defined as a UL1 award with a linked KL2 award, this term was added to the section.
Pilot awards	This phrase related to CTSA-funded institutional grants designed to innovate clinical and translational science ideas. Pilot awards are grants specifically designed to generate preliminary data needed to apply for research grants; improve clinical design, biostatistics, clinical research ethics, informatics or regulatory pathways; develop new technologies; and provide a collaborative approach to research.
TL1	This abbreviation is an NIH-funded award that supports research training experiences for predoctoral trainees who are interested in pursuing research careers in multidisciplinary clinical and translational science.

This table provides the categories and terms searched and identified for CTSA Search Solutions website. COVID-19: Sars-CoV-2; CTSA: Clinical and Translational Science Awards; NCATS: National Center for the Advancement of Tranclational Science; BERD: Biostatistics, Epidemiology, and Research Design; KL2: NIH linked mentored career development award; NIH: National Insitute of Health; UL1: NIH linked specialized center cooperative agreement; TL1: NIH linked training award.

### Aim 2: Collection of CTSA Website URLs

**Fig. 1.**
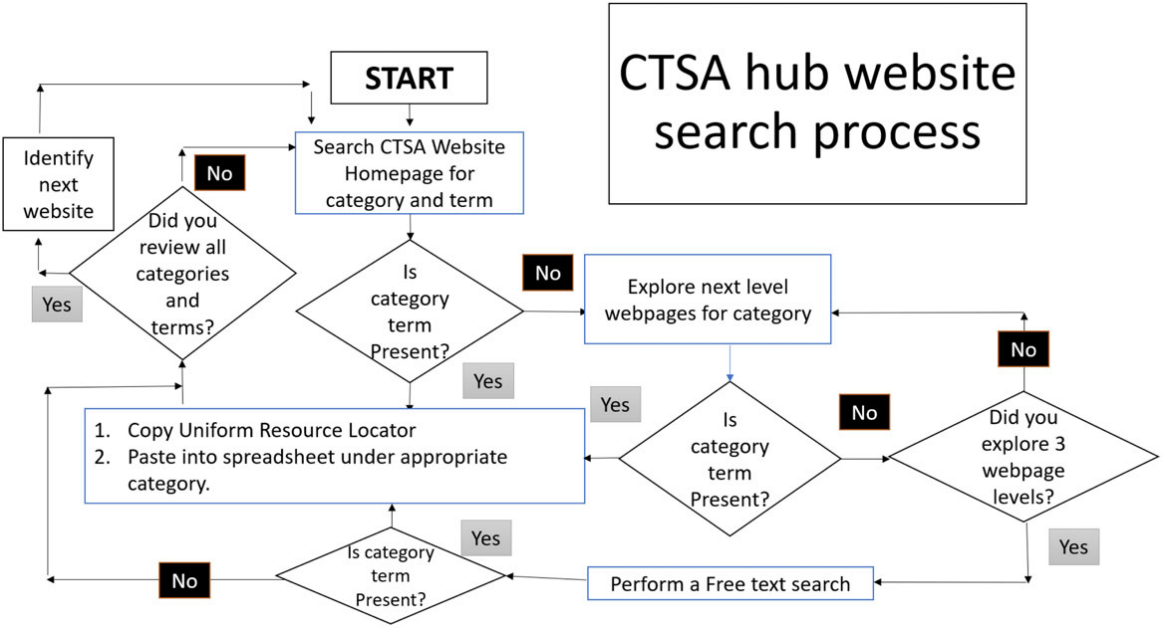
CTSA hub website search process. This is a flow diagram of the search process for the categories and terms in Table 1. CTSA: Clinical and Translational Science Award.

To collect URLs that align with the structured search terms, a systematic website search protocol was developed to manually scrub CTSA hub websites for webpages on the topics identified in Aim 1 (Fig. 1: CTSA hub website search process). Each CTSA hub from the list generated in Aim 1 was searched via an internet search engine for the CTSA hub home page. Once the CTSA home page was identified, the website navigation headers and sidebars were explored for webpages that included the topics identified in Aim 1. In the event the data collector was unable to identify a topic through the navigation headers and sidebars, then a second search process was conducted through free-text search in the CTSA hub website’s search box, if such a search box existed.

In the event a webpage or substantive content about the search term was identified, the URL was copied and pasted into a CTSA Search Solutions MS Excel spreadsheet. Once all searched terms were identified and collected from all CTSA hubs from the generated list, then a second team member reviewed the links for validation. Any discrepancies during validation were documented, discussed, and resolved via group consensus. The most common discrepancy was that the initial data collector labeled a topic as not existing for a particular CTSA hub website, but then the validator was able to find content for that topic.

### Aim 3: Create, Preserve, and Disseminate Content Within a Website

**Fig. 2.**
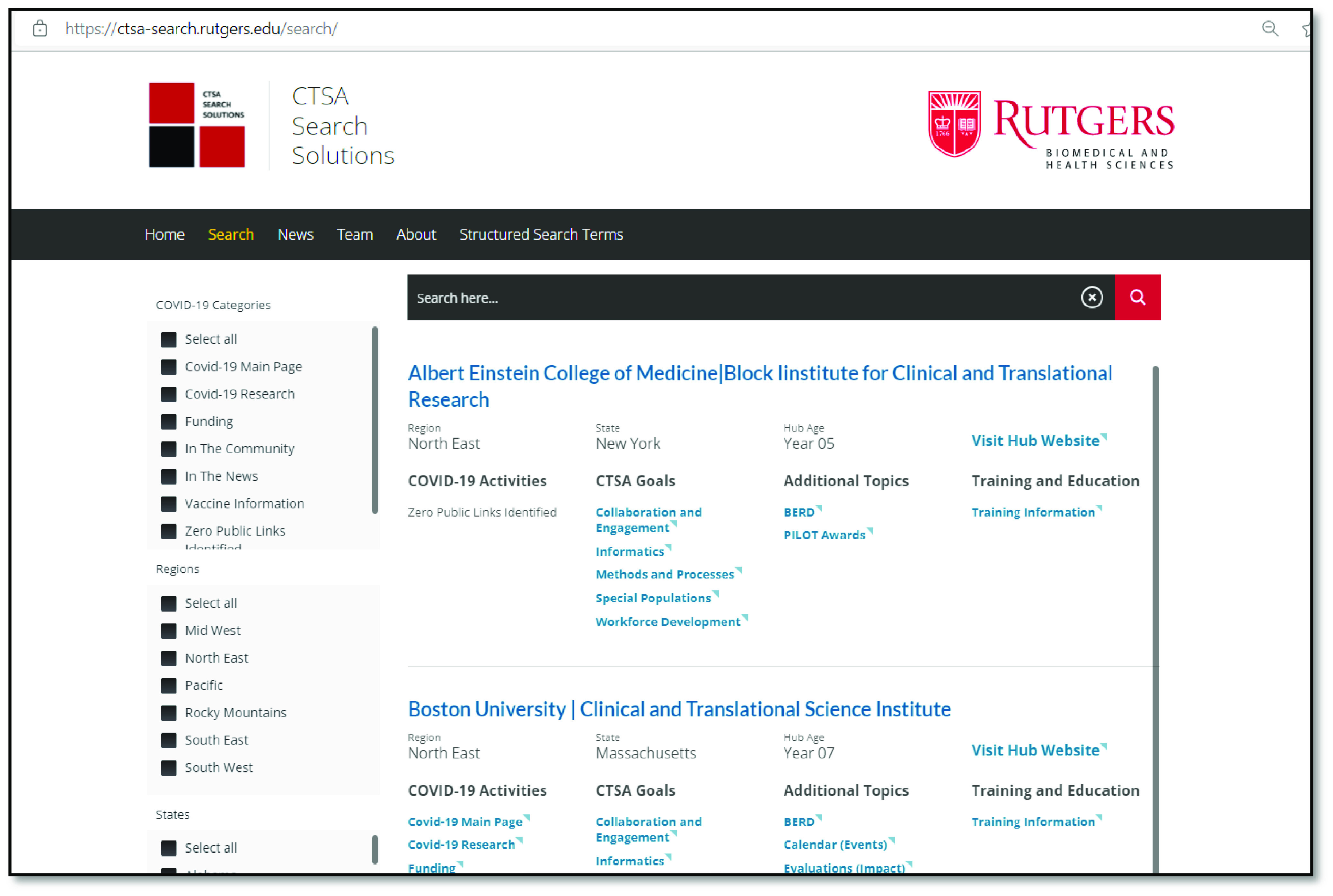
CTSA Search Solutions search page screenshot. This is a screenshot of the searchable, online database at the CTSA Search Solutions website. CTSA: Clinical and Translational Science Award; COVID-19: Sars-Cov-2; BERD: Biostatistics, Epidemiology, and Research Design.

The comprehensive collection of URLs was built into a database with a front-facing website called CTSA Search Solutions (Fig. 2: CTSA Search Solutions search page screenshot). Web Consulting Services (WCS), a web agency within Rutgers University, was contracted to develop and design the website and database with the goal of providing the simplest user interface possible within the parameters of the available content management system, WordPress. The WCS developers extended WordPress to include custom functionalities for populating the Search page with information for each hub, segmented by different taxonomies, such as region, state, CTSA goals, hub age, and COVID-19 categories. Once the website and database were completed, research assistants conducted independent reviews of functionality and validation of links in the Summer and Fall of 2021.

### Aim 4: Beta Testing

In November 2021, the CTSA Search Solutions website platform was introduced to two key user groups CTSA wide, the CLIC Communicators and CLIC Evaluators Groups. At the national CLIC meetings, CTSA hub communicators and evaluators were introduced to the website platform and provided an access link to pilot test and review their own hub information. They were also provided a survey for their feedback. Twelve one-on-one, follow-up interviews were conducted with volunteers from these groups (all from different hubs) to beta test the website platform and provide more detailed feedback on: (1) how the database would be used; (2) which other types potential users would find this platform helpful for their CTSA role; (3) any additional information that would be helpful to include in the database or on the website; and (4) improvements on user experience. Suggestions from the user interviews that were feasible to implement were implemented into the system before the public launch of the website.

## Results

### User Feedback from Beta Testing

During the user interviews, participants were asked what potential use the database had in their workflow. The most common uses described were to: (1) understand more about programs, services, and initiatives at other hubs; (2) identify potential collaborators, specifically for combining strengths to submit proposals for NCATS funding opportunities; (3) develop ideas for updating hub website content and aligning it with the rest of the CTSA hub network; and (4) assist the research faculty at their home institutions in understanding the opportunities available to them through CTSA hubs. Participants identified administrators and core leads as users who might find this database and web platform helpful to their role at the CTSA hub. Participants also suggested adding links to social media channels, hub contact pages, identification of team leaders, and a suggestion box feature for recommendations for new content or for content that needs updating. User improvement suggestions focused mostly on improvements to the functionality of the search filters and the ability to share/save search results.

### Current Product

CTSA Search Solutions can be found at https://ctsa-search.rutgers.edu/. The database includes access to 63 CTSA hub websites with 87 structured search term options and over 800 links collected, organized, and published. Hub content can be filtered by state, region, or number of years funded, CTSA goals, topics under CTSA common cores and initiatives, and COVID-19 activities to make detailed comparisons with the data identified. Users can also perform free-text searches of hub names or portions of hub names in the search bar. The home page for every hub can be accessed directly from the search page. CTSA Search Solutions also includes an individual summary page for each hub that includes both general content about the hub and specific information about its educational offerings based on the five training categories described in Aim 1.

## Discussion

CTSA Search Solutions is intended to allow for a wide breadth of CTSA personnel –communicators, evaluators, core leads, administrators, and researchers – to: (1) identify job-related CTSA hub program content and (2) conduct research into program hub outputs across the nationwide CTSA hub network.

On the most practical level, CTSA Search Solutions can help core leads determine common best practices, and it can help communicators design better website content to represent the value and opportunities brought by CTSA hubs. It can also be a source of information to conduct research into hub function and output. For example, since its launch, CTSA Search Solutions has been used to support research related to: (1) an improved understanding of best practices for CTSA Pilot Awards; (2) COVID-19 vaccine community outreach practices among the CTSA hub network during the height of the pandemic; (3) education and training programs among CTSA hubs; and (4) the prevalence of social media use among CTSA hubs [11–14]. On an aspirational level, CTSA Search Solutions has the potential to be another option for benchmarking CTSA hubs. For example, with further functionality development and refinement, it could be used to help develop a maturity model through an assessment of stratified CTSA hub website content and themes across hubs from their first cycle through to those in their third cycle or beyond. Overall, the opportunities presented by CTSA Search Solutions have the potential to support a variety of activities across the CTSA hub network.

CTSA Search Solutions in its current form is a proof of concept for a more robust tool that is envisioned. Next steps for this project include: (1) expansion of the database search parameters and content, primarily based on the user feedback from our beta testing in November 2021; (2) implementation of user improvement recommendations, as identified during beta testing; (3) developing a sustainable process for updating content as CTSA hub websites change and evolve; and (4) integrating the new NCATS goals established in the UM1 model and being adaptable to any future evolution of NCATS. One sustainability model under consideration is a hybrid model that would have dedicated personnel for validating and updating database content at regular intervals throughout the year with a component that would also allow CTSA hub representatives to submit updates and/or corrections at any time. However, this model would require additional funding and resources. Other future activities that the co-investigators would like to explore include: (1) provisions for data visualization functionalities that can help users understand CTSA hub website content metrics both individually and as a network and (2) collaboration with CTSA community engagement groups to understand if CTSA Search Solutions would be valuable to patient or research participant communities and, if so, how best to disseminate this resource to those groups. Existing funding for CTSA Search Solutions will keep the website live and allow for quarterly platform validation and updates until July 2023. The project team is pursuing follow on funding and collaboration opportunities that will allow this database to be sustainably maintained and updated beyond 2023, as well as to pursue the aforementioned next steps and future activities.
